# Therapeutic outcome of patients with Lennox–Gastaut syndrome with mitochondrial respiratory chain complex I deficiency

**DOI:** 10.3389/fneur.2024.1305404

**Published:** 2024-03-11

**Authors:** Ji-Hoon Na, Young-Mock Lee

**Affiliations:** Department of Pediatrics, Gangnam Severance Hospital, Yonsei University College of Medicine, Seoul, Republic of Korea

**Keywords:** epilepsy, mitochondrial disease, mitochondrial dysfunction, mitochondrial respiratory chain complex I deficiency, Lennox–Gastaut syndrome (LGS)

## Abstract

**Background:**

Lennox–Gastaut syndrome (LGS), a severe developmental epileptic encephalopathy, has various underlying causes. Mitochondrial respiratory chain complex I (MRC I) deficiency is an important cause of metabolic disorders such as mitochondrial dysfunction that can compromise brain function, thereby causing intractable epilepsy, including LGS. Thus, it can be expected that the presence or absence of MRC I deficiency may affect the treatment outcome of patients with LGS.

**Objectives:**

In this retrospective study, we aimed to investigate differences in the epilepsy characteristics and treatment outcomes between patients with LGS with and without MRC I deficiency.

**Methods:**

We retrospectively reviewed the medical records of 92 patients with LGS. We divided 68 patients with LGS according to the presence (*n* = 30) or absence (*n* = 38) of MRC I deficiency and compared their epilepsy characteristics.

**Results:**

Generalized tonic and drop seizures were significantly worse in patients with LGS and MRC I deficiency than in those without MRC I deficiency group at the 1-year follow-up (*p* < 0.001) and final follow-up 1 (*p* < 0.001). Patients with LGS and MRC I deficiency had significantly fewer electroencephalogram (EEG) improvements compared to those without MRC I deficiency at the 1-year follow-up (*p* = 0.031). Additionally, in the final follow-up period, patients with LGS and MRC I deficiency had significantly less improvement in EEG findings compared to patients without MRC I deficiency (*p* < 0.001).

**Conclusion:**

The overall treatment prognosis—in terms of improvement in traumatic generalized tonic seizure, drop seizure, and EEG findings—is worse in patients with LGS and MRC I deficiency than that in patients with LGS but without MRC I deficiency. Additional and targeted treatment is required to treat LGS with MRC I deficiency.

## 1 Introduction

Lennox–Gastaut syndrome (LGS) is a rare type of intractable epilepsy and developmental epileptic encephalopathy (DEE) characterized by a triad of symptoms: refractory seizures, progressive cognitive impairment, and severe electroencephalogram (EEG) abnormalities (1–3). Various underlying causes, including genetic, metabolic, and structural factors, can lead to LGS. Although knowledge about the diagnosis, treatment, and prognosis of LGS has evolved through decades of research, the clinical management of LGS remains challenging (4–7).

Parallel to the rapid development of etiologic tests, studies (7, 8) have focused on developing and optimizing treatment strategies guided by the etiology of LGS. Various underlying causes have been associated with severe LGS; therefore, the therapeutic outcome of patients varies based on the cause (9, 10). One underlying cause is mitochondrial dysfunction, such as mitochondrial respiratory chain complex I (MRC I) deficiency, which is an important metabolic factor that can affect multiple major organs and is a potential therapeutic target. MRC I deficiency is the most common cause of mitochondrial dysfunction (7, 11, 12). Mitochondria are extensively distributed in the brain; therefore, MRC I deficiency could cause intractable epilepsy such as status epilepticus by severely reducing brain function (13–15). In turn, MRC I deficiency strongly affects the treatment and prognosis of intractable epilepsy syndromes such as LGS; hence, some changes in the treatment strategy for LGS may be required, depending on whether MRC I deficiency is involved (16, 17).

Thus, the purpose of this study was to examine differences in epilepsy characteristics and treatment outcomes between patients with LGS with or without MRC I deficiency. Our findings provide a valuable reference for epileptologists treating LGS and demonstrate the need for targeted LGS therapy that can modulate MRC I deficiency for better patient outcomes.

## 2 Methods

### 2.1 Patients

**Figure 1:**
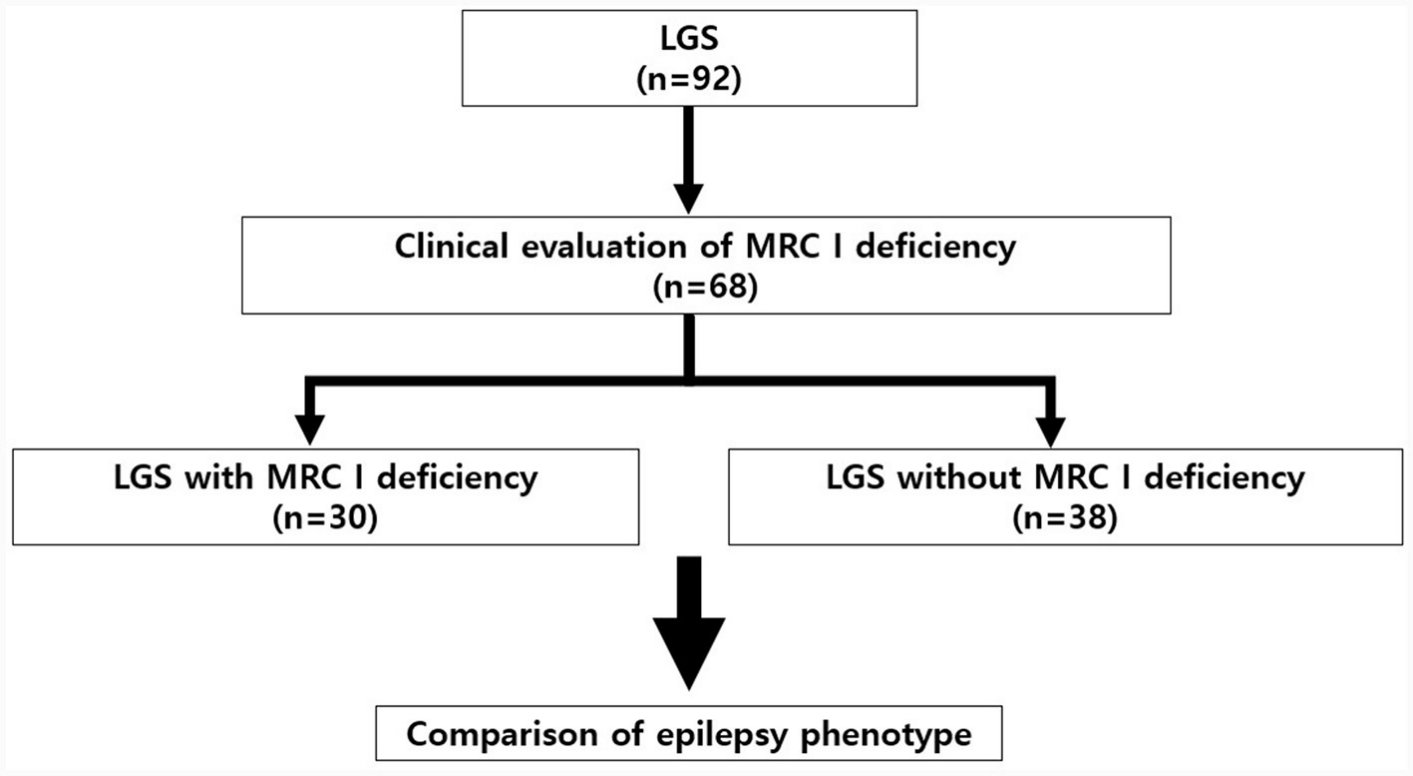
Study design and patient selection protocol. LGS, Lennox–Gastaut syndrome; MRC, mitochondrial respiratory chain complex.

We retrospectively reviewed the medical records of 92 patients with LGS who underwent treatment at Gangnam Severance Children's Hospital (Seoul, Republic of Korea) between 2010 and 2022. The diagnostic criteria for LGS were as follows: (1) the presence of multiple types of seizures, including generalized tonic seizures combined with drop seizure, myoclonic, atonic, atypical absence, and focal seizures; (2) severe EEG abnormalities, including generalized paroxysmal fast activity (GPFA) and diffuse slow spike-wave complexes during wake or sleep; and (3) progressive cognitive impairment (1, 17). The presence or absence of MRC I deficiency was clinically evaluated in patients with LGS. Of the 92 patients with LGS, 68 patients were tested for MRC I deficiency. Based on the results, we divided these patients into those with MRC I deficiency (*n* = 38) and those without (*n* = 30) (Figure 1). All patients were treated according to the standard protocol for LGS (6, 10). However, antiseizure medications (ASMs) that may cause MRC I deficiency such as valproic acid were not prescribed for patients with LGS and MRC I deficiency (14, 17).

### 2.2 Evaluation of patients with LGS

We retrospectively evaluated the EEG and magnetic resonance imaging (MRI) findings, seizure type, status epilepticus history, number of anti-seizure medications (ASMs) ever used, history of diet therapy or epilepsy surgery, and seizure reduction rate at the 1-year and final follow-ups for all patients. Because patients with LGS have a slower response to anti-seizure medication compared to general epilepsy, have extremely rare seizure-freedom, and have many disabling seizures, we believe that a more convenient and detailed seizure classification is needed. Therefore, the seizure classification of patients in this study used the quadrant method, referring to Engel's classification. Seizure reduction was evaluated through regular follow-up by dedicated guardians and medical staff, and was recorded by referring to daily seizure diaries. “Reduction rate 100%” means seizure-freedom, and “Reduction rate 0%” means no response to treatment at all (17, 18). Of the 68 patients, 42 patients consented to genetic testing by targeted exome sequencing to investigate the possible genetic cause of LGS.

### 2.3 Evaluation of MRC I deficiency

MRC I deficiency was confirmed if MRC I defects were identified through the biochemical evaluations. Muscle biopsy specimens were processed, using standard staining methods, based on periodic acid–Schiff, modified Gomori trichrome, adenosine triphosphatase, pH 9.4, nicotinamide adenine dinucleotide tetrazolium reductase, and succinate dehydrogenase. Based on light microscopic findings, ragged red fibers were included as an indicator of mitochondrial dysfunction, but non-specific findings such as type I or II atrophy were excluded. Moreover, all specimens were examined for changes such as mitochondrial pleoconia and megaconia. Finally, MRC enzyme complex activities were evaluated using standard spectrophotometric assays to assess the activities of the nicotinamide adenine dinucleotide and hydrogen–coenzyme Q (CoQ) reductase (complex I), succinate–CoQ reductase (complex II), succinate–cytochrome c reductase (complex II–III), cytochrome c reductase (complex III), cytochrome c oxidase (complex IV), and citrate synthase by using mitochondria isolated from freshly prepared muscle tissues (17–19). We defined an MRC defect as a reduction in residual enzyme activity to < 10% of that of the controls (20, 21). To evaluate system and organ involvement at the time of LGS diagnosis, all systems and organs in which MRC I deficiency could occur were considered, including the central nervous system (e.g., seizure, developmental delay or regression), muscles (e.g., muscle weakness, exercise intolerance, low muscle tone), endocrine system (e.g., diabetes mellitus, recurrent pancreatitis), gastrointestinal system (e.g., gastroesophageal reflux disease, swallowing difficulties, motility disorders), respiratory system (e.g., breathing difficulties, recurrent pneumonia, difficulty in effective coughing), eyes (e.g., optic atrophy, ptosis), heart (e.g., arrythmia, cardiomyopathy), kidneys (e.g., tubular acidosis), ears (e.g., hearing problems), and hematologic system (11, 17–19).

### 2.4 Neuroimaging

At the time of diagnosis, all patients underwent brain MRI, and some patients also underwent brain magnetic resonance spectroscopy (MRS) (19). Using the MRI and MRS data, we compared the structural and metabolic status between the two groups.

### 2.5 Evaluation of treatment outcomes

We evaluated seizure reduction during the follow-up period. The initial EEG, 1-year follow-up EEG, and last follow-up EEG findings were evaluated. The EEG findings were graded as follows: 1, normal; 2, slow and disorganized background rhythm without focal or unilateral sharp wave discharges; 3, slow and disorganized background rhythm with focal or unilateral sharp wave discharges; and 4, slow and disorganized background rhythm with multifocal sharp wave discharges with generalized slow spike-waves (GSSW) and GPFA (7, 18). EEG findings before and after treatment were evaluated and compared. Changes in the rate of all seizures, the rate of generalized tonic and drop seizures, and EEG findings at the 1-year and last follow-ups after treatment were compared between the two groups.

### 2.6 Statistical analyses

All statistical analyses were conducted using R (version 4.2.2; R Foundation for Statistical Computing, Vienna, Austria). Continuous variables are presented as the median and range (minimum–maximum), and ratios for each group are expressed as percentages. Parametric comparisons were conducted by using Student's *t*-test. Before starting the parametric test, the related data were confirmed to be parametric through a normality test (Kolmogorov-Smirnov test). The chi-squared or Fisher's exact test was used for categorical variables. Results were statistically significant at *p* < 0.05.

## 3 Results

### 3.1 General characteristics of the patients

The study included 39 (57.4%) male patients, 17 (56.7%) of whom had LGS with MRC I deficiency. Prematurity, perinatal asphyxia, hypoxic brain damage, and neonatal seizure were observed in 4.4%, 13.2%, 8.8%, and 10.3%, of patients, respectively. The age of first seizure onset was < 2 years in both groups. The median age at the LGS diagnosis was 5 years, and the median interval between the first seizure and the LGS diagnosis was 3 years. The follow-up period ranged from 1 year to 16 years, with a median of 6 years across all patients (Table 1).

**Table 1:** General characteristics of patients enrolled in this study.

	**Total**	**LGS with MRC I deficiency**	**LGS without MRC I deficiency**
	**(*****n** =* **68)**	**(*****n** =* **30)**	**(*****n** =* **38)**
**Sex (male, %)**	39 (57.4)	17 (56.7)	22 (57.9)
**Neonatal history, n (%)**			
Premature birth	3 (4.4)	1 (3.3)	2 (5.3)
Perinatal asphyxia	9 (13.2)	4 (13.3)	5 (13.2)
Hypoxic brain damage	6 (8.8)	2 (6.7)	4 (10.5)
Neonatal seizure	7 (10.3)	2 (6.7)	5 (13.2)
**Age at the time of the first seizure (years)**			
< 2, n (%)	51 (75.0)	24 (80.0)	27 (71.1)
≥2, n (%)	17 (25.0)	6 (20.0)	11 (29.9)
**Age at the time of the LGS diagnosis, years, median (range)**	5 (1–20)	6 (2–19)	4 (1–20)
**Interval between the first seizure and LGS diagnosis, years, median (range)**	3 (0–18)	3 (0–17)	2 (0–18)
**Follow-up period, years, median (range)**	6 (2–16)	8 (2–16)	4 (2–15)

LGS, Lennox–Gastaut syndrome; MRC I, mitochondrial respiratory chain complex I.

### 3.2 Mitochondrial characteristics of patients with LGS and MRC I deficiency

To identify the mitochondrial characteristics of patients with LGS and MRC I deficiency, organ involvement, serum lactic acid level, and muscle biopsy findings were investigated. Central nervous system and muscle dysfunction were present in all patients. Endocrinological (83.3%), gastrointestinal (80.0%), respiratory (56.7%), heart (46.7%), eye (46.7%), and kidney (46.7%) dysfunctions were also present in these patients. At the time of LGS diagnosis, the serum lactic acid level was mildly increased in most (70%) patients. Under the light microscope, 43.3% of muscle biopsy specimens from patients with LGS and MRC I deficiency showed mitochondrial-specific findings, and under the electron microscope, 46.7% of specimens showed pleoconia or megaconia. Biochemical analysis revealed MRC I deficiencies in all patients with LGS and MRC I deficiency (Table 2).

**Table 2:** Mitochondrial characteristics of patients with LGS and MRC I deficiency.

	**LGS wit MRC I deficiency**
	**(*****n** =* **30)**
**Organ involvement**, ***n*** **(%)**	
Neuromuscular system	30 (100)
Endocrine system	25 (83.3)
Gastrointestinal tract	24 (80.0)
Respiratory system	17 (56.7)
Heart	14 (46.7)
Eyes	14 (46.7)
Kidneys	14 (46.7)
Ears	10 (33.3)
Hematology	3 (10.0)
**Grading of the serum lactic acid level**, ***n*** **(%)**	
Normal	8 (26.7)
Mildly increased (< 2-fold)	21 (70.0)
Moderately increased (2- to 3-fold)	1 (3.3)
Severely increased (>3-fold)	0
**Muscle biopsy findings, n (%)**	
**Light microscopy**	
Normal	14 (46.7)
Mitochondrial-specific finding	13 (43.3)
Mitochondrial non-specific finding	3 (10.0)
**Electron microscopy**	
Normal	16 (53.3)
Pleoconia only	4 (13.3)
Megaconia only	8 (26.7)
Pleoconia + megaconia	2 (6.7)
**MRC defect, n (%)**	
MRC I deficiency	30 (100)

LGS, Lennox–Gastaut syndrome; MRC I, mitochondrial respiratory chain complex I.

### 3.3 Brain imaging findings

MRI revealed white matter, cerebellar, and cerebral atrophy in both groups. Of note, cerebral atrophy was significantly greater in patients with LGS and MRC I deficiency than in those with LGS without MRC I deficiency (80% vs. 44.7%, *p* = 0.006). MRS revealed a lactate peak in 16.7% of patients with LGS and MRC I deficiency, but this peak was absent in patients with LGS without MRC I deficiency (*p* = 0.006). In addition, a decreased *N*-acetylaspartate peak was observed in a significantly greater number of patients with LGS and MRC I deficiency than in those without MRC I deficiency (76.7% vs. 18.4%, *p* < 0.001) (Table 3).

**Table 3:** Brain imaging analysis.

	**Total**	**LGS with MRC I deficiency**	**LGS without MRC I deficiency**	***p*-value**
	**(*****n** =* **68)**	**(*****n** =* **30)**	**(*****n** =* **38)**	
**Brain MRI lesion**				
Basal ganglia	6 (8.8)	4 (13.3)	2 (5.3)	0.394
Thalamus	6 (8.8)	5 (16.7)	1 (2.6)	0.080
Midbrain	3 (4.4)	2 (6.7)	1 (2.6)	0.579
Pons	1 (1.5)	1 (3.3)	0	0.441
Medulla	1 (1.5)	1 (3.3)	0	0.441
Cerebral cortex	9 (13.2)	3 (10.0)	6 (15.8)	0.721
White matter	30 (44.1)	15 (50.0)	15 (39.5)	0.464
Cerebellum	13 (19.1)	8 (26.7)	5 (13.2)	0.217
Atrophy	41 (60.3)	24 (80.0)	17 (44.7)	0.006
**Brain MRS findings**				
Lactate peak (+)	5 (7.4)	5 (16.7)	0	0.014
Decreased NAA peak (+)	30 (44.1)	23 (76.7)	7 (18.4)	< 0.001

The data are presented as n (%).

LGS, Lennox–Gastaut syndrome; MRC I, mitochondrial respiratory chain complex I; MRI, magnetic resonance imaging; MRS, magnetic resonance spectroscopy; NAA, N-acetylaspartate.

### 3.4 Epilepsy characteristics of enrolled patients

The median age of seizure onset was 1 year in both groups. For both groups, the seizure type primarily included generalized tonic seizure and drop seizure, although other seizure types such as myoclonic, atonic, atypical absence, and focal seizures were also observed. Approximately 10% of patients with LGS and MRC I deficiency experienced status epilepticus, and a similar trend was noted in those without MRC I deficiency. The number of ASMs ever used was significantly higher in patients with LGS and MRC I deficiency than in those without MRC I deficiency. Approximately 50% of patients in both groups had a history of diet therapy. One patient with LGS and MRC I deficiency and six patients with LGS without MRC I deficiency had undergone epilepsy surgery in the past. Changes in seizure frequency did not differ significantly between groups at the 1-year and final follow-up timepoints. However, at the last follow-up, patients with LGS without MRC I deficiency showed improved distribution of seizure reduction rates compared to those with MRC I deficiency. Moreover, generalized tonic and drop seizures had significantly improved in patients with LGS without MRC I deficiency compared to those with MRC I deficiency at the 1-year follow-up (*p* < 0.001) and final follow-up (*p* < 0.001).

**Figure 2:**
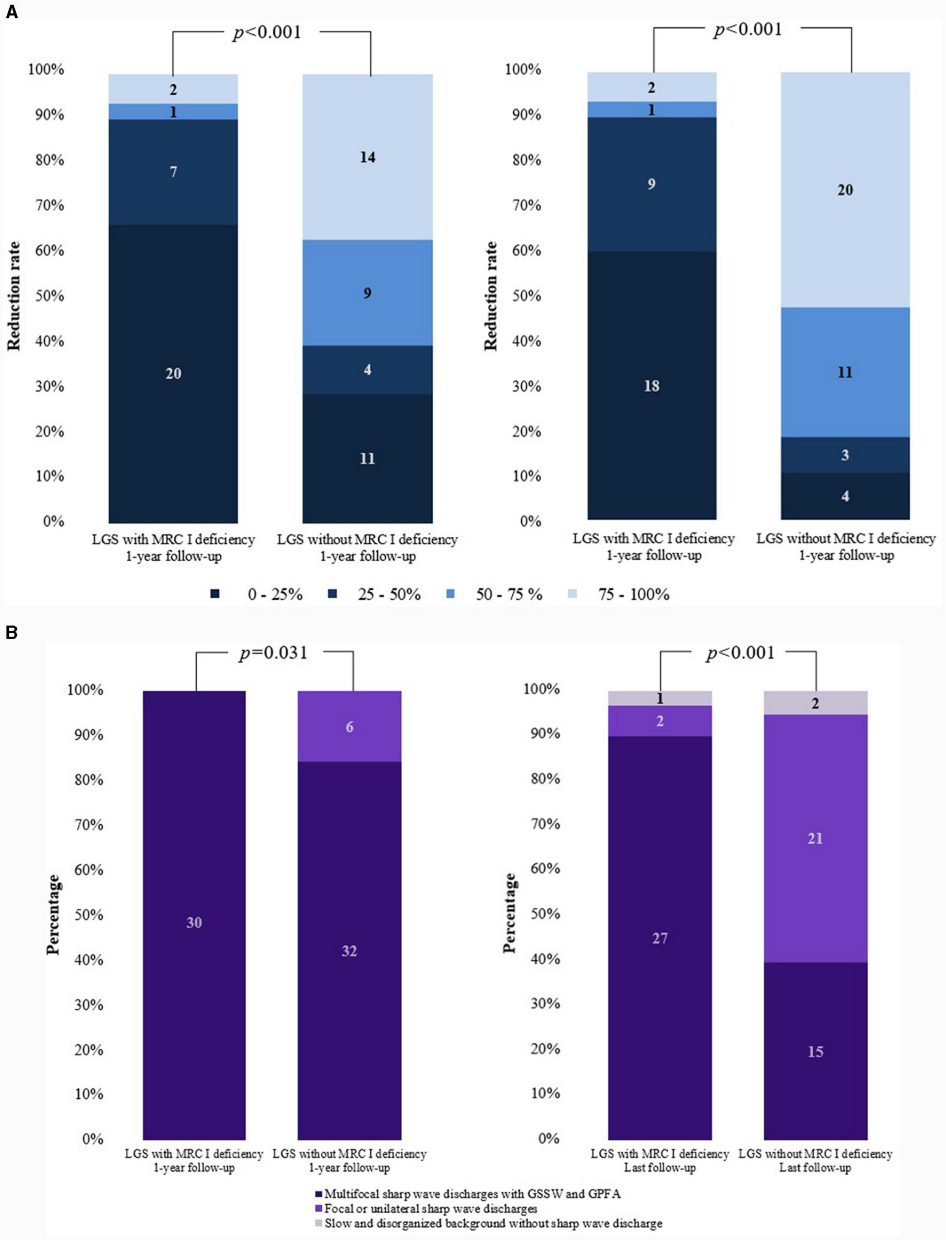
Comparisons of changes in seizure frequency and EEG findings. **(A)** Reduction in the rate of generalized tonic and drop seizures. **(B)** Changes in EEG findings. EEG, electroencephalogram; LGS, Lennox–Gastaut syndrome; MRC, mitochondrial respiratory chain complex; GSSW, generalized slow spike and wave; GPFA, generalized paroxysmal fast activity.

Among the 42 patients who underwent genetic testing, pathogenic variants were found in 26.2% of patients. In the patients with LGS and MRC I deficiency, pathogenic variants of m.8993T>G, m.10191T>C, *SLC6A8, CACNA1A, ALG13*, and *POLR3B* were identified. In the patients with LGS without MRC I deficiency, pathogenic variants of *KCNQ2, SCN2A, ASXL2*, and *KCNH2* were identified (Table 4, Figure 2A).

**Table 4:** Epilepsy characteristics of the patients.

	**Total**	**LGS with MRC I deficiency**	**LGS without MRC I deficiency**	***p*-value**
	**(*****n** =* **68)**	**(*****n** =* **30)**	**(*****n** =* **38)**	
**Seizure onset age, years, median (range)**	1 (0–7)	1 (0–7)	1 (0–5)	0.630
**Seizure type**, ***n*** **(%)**				
Generalized tonic and drop seizure	64 (94.1)	30 (100)	38 (100)	0.861
Myoclonic	15 (22.1)	8 (26.7)	7 (18.4)	
Atonic	17 (25.0)	7 (23.3)	10 (26.3)	
Atypical absence	4 (5.9)	1 (3.3)	3 (7.9)	
Focal	16 (23.5)	8 (26.7)	8 (21.1)	
**Status epilepticus**, ***n*** **(%)**	6 (8.8)	3 (10.0)	3 (7.9)	0.544
**Number of anti-seizure medications ever used, median (range)**	4 (1–8)	4.5 (2–8)	3 (1–6)	0.005
**History of diet therapy**, ***n*** **(%)**				
Classic KD (4:1, 3:1)	19 (27.9)	10 (33.3)	9 (23.7)	0.300
MAD	11 (16.2)	4 (13.3)	7 (18.4)	
LGIT (low glycemic index)	2 (2.9)	0	2 (5.3)	
**History of epilepsy surgery**, ***n*** **(%)**				
Corpus callosotomy	3 (4.4)	1 (3.3)	2 (5.3)	0.459
VNS insertion	1 (1.5)	0	1 (2.6)	
Resective surgery	3 (4.4)	0	3 (7.9)	
**Change in seizure frequency**, ***n*** **(%)**				
**Reduction rate of all seizures at the 1-year follow-up**				
0–25%	22 (32.4)	9 (30.0)	13 (34.2)	0.704
25–50%	10 (14.7)	6 (20.0)	4 (10.5)	
50–75%	6 (8.8)	3 (10.0)	3 (7.9)	
75–100%	30 (44.1)	12 (40.0)	18 (47.4)	
**Reduction rate of all seizures at the last follow-up**				
0–25%	12 (17.6)	7 (23.3)	5 (13.2)	0.481
25–50%	15 (22.1)	7 (23.3)	8 (21.1)	
50–75%	5 (7.4)	3 (10.0)	2 (5.3)	
75–100%	36 (52.9)	13 (43.3)	23 (60.5)	
**Reduction rate of generalized tonic and drop seizures at the 1-year follow-up**				
0–25%	31 (45.6)	20 (66.7)	11 (28.9)	< 0.001
25–50%	11 (16.2)	7 (23.3)	4 (10.5)	
50–75%	10 (14.7)	1 (3.3)	9 (23.7)	
75–100%	16 (23.5)	2 (6.7)	14 (36.8)	
**Reduction rate of generalized tonic and drop seizures at the last follow-up**				
0–25%	22 (32.4)	18 (60.0)	4 (10.5)	< 0.001
25–50%	12 (17.6)	9 (30.0)	3 (7.9)	
50–75%	12 (17.6)	1 (3.3)	11 (28.9)	
75–100%	22 (32.4)	2 (6.7)	20 (52.6)	
**Genetic test results, n (%)**	(N=42)			0.475
Pathogenic variant	11 (26.2)	7 (16.7)	4 (9.5)	
Negative finding	31 (73.8)	13 (31.0)	18 (42.9)	

LGS, Lennox–Gastaut syndrome; MRC I, mitochondrial respiratory chain complex I; KD, ketogenic diet; MAD, modified Atkins diet; LGIT, low glycemic index treatment; VNS, vagus nerve stimulation.

### 3.5 EEG findings of enrolled patients

Differences in EEG findings were examined between the groups at the 1-year and final follow-ups. At diagnosis, the initial EEG findings showed multifocal sharp waves with GSSW and GPFA in both groups. There was no difference in the EEG findings of patients with LGS and MRC I deficiency at the 1-year post-treatment follow-up compared with the pretreatment findings. However, some patients with LGS without MRC I deficiency showed significantly greater GSSW and GPFA EEG improvements than those with MRC I deficiency (*p* = 0.031). At the final follow-up, EEG findings improved for both groups, but patients with LGS without MRC I deficiency had a significantly greater improvement than those with MRC I deficiency (*p* < 0.001) (Table 5, Figure 2B).

**Table 5:** EEG findings of the patients.

	**Total**	**LGS with MRC I deficiency**	**LGS without MRC I deficiency**	***p*-value**
	**(*****n** =* **68)**	**(*****n** =* **30)**	**(*****n** =* **38)**	
**Initial**, ***n*** **(%)**				
Normal	0	0	0	-
Slow and disorganized	0	0	0	
Focal or unilateral sharp	0	0	0	
Multifocal sharp with GSSW and GPFA	68	30	38	
**1-year follow-up**, ***n*** **(%)**				
Normal	0	0	0	0.031
Slow and disorganized	0	0	0	
Focal or unilateral sharp	6	0	6	
Multifocal sharp with GSSW and GPFA	60	30	32	
**Last follow-up**, ***n*** **(%)**				
Normal	0	0	0	< 0.001
Slow and disorganized	3	1	2	
Focal or unilateral sharp	23	2	21	
Multifocal sharp with GSSW and GPFA	42	27	15	

EEG, electroencephalogram; LGS, Lennox–Gastaut syndrome; MRC I, mitochondrial respiratory chain complex I; GSSW, generalized slow spike and wave; GPFA, generalized paroxysmal fast activity.

## 4 Discussion

LGS is an epileptic encephalopathy with varied etiologies; however, etiology-specific treatments have not yet been identified. Therefore, most patients with LGS are treated according to a generalized protocol, based on the expert opinion and clinical experience of epileptologists (6, 10). In this study, patients with LGS were divided based on the status of MRC I deficiency and their differential therapeutic outcomes were compared. Our results showed that the overall treatment prognosis, in terms of improved generalized tonic and drop seizures and EEG findings, was worse in patients with LGS and MRC I deficiency than in patients with LGS without MRC I deficiency.

The recent trend in LGS treatment has been to treat based on etiology. Whole-exome sequencing is useful for elucidating the genetic etiologies of LGS. Pathogenic variants of *CDKL5, KCNQ2, STXBP1, SCN1A, SCN2A, SCN8A, ALG13, GABRB3, TSC1*, and *TSC2* are considered the main causes of LGS, and corresponding gene-targeted therapies are being developed (3, 22). When ASMs are used for LGS treatment, the use of a sodium-channel blocker is recommended for LGS with polymorphism in sodium or potassium channels attributed to variations in *SCN2A, SCN8A*, and *KCNQ2* (23). The safety and efficacy of ganaxolone was recently verified for treating CDKL5-related DEE (24). A study (25) has shown that a ketogenic diet is the most effective in maintaining a seizure-free status in STXBP1-related DEE. For tuberous sclerosis complex-related LGS, everolimus and cannabidiol can modulate the mammalian target of rapamycin (mTOR) pathway, and vigabatrin has been confirmed as a preventive treatment (26). Thus, to devise novel LGS treatment strategies, scientists should focus on identifying the relationship between genotype and phenotype, analyzing LGS etiology mechanisms, and exploring targeted therapies.

Mitochondrial dysfunction, such as MRC I deficiency, is also an important etiology of LGS and can be targeted for treatment (11, 27). Although many pathogenic variants of mitochondrial DNA have been identified, pathogenic variants of nuclear DNA associated with MRC I deficiency have yet to be identified (11, 15). MRC I deficiency, whether inherited or acquired, causes oxidative stress, the disruption of calcium homeostasis, decreased neuronal plasma membrane potentials, and network inhibition due to reactive oxygen species (ROS). These factors can collectively increase neuronal excitability and result in intractable seizures (13–15). The pathogenic variants of m.8993T>G, m.10191T>C, and *SLC6A8* found in patients with LGS and MRC I deficiency in our study have been related to MRC I deficiency (15, 28). Further functional genetic studies are needed to determine whether *CACNA1A* and *ALG13*, which are genes associated with DEE, and *POLR3B*, which is associated with demyelinating Charcot–Marie–Tooth disease type 1, are associated with MRC I deficiency. Overall, the findings of the present study demonstrated that the treatment response of patients with LGS and MRC I deficiency was poorer than that of those without MRC I deficiency, thereby suggesting that MRC I deficiency negatively impacts therapeutic outcomes in LGS. Therefore, future research studies that focus on identifying novel targeted LGS treatment methods and agents that can improve outcomes for patients with LGS and MRC I deficiency are imperative.

MRC I deficiency is treated with a cocktail of ubiquinone, high-dose riboflavin, thiamine, niacin, biotin, and L-carnitine. This approach has been widely applied for a long time and partially improves MRC I deficiency; however, its efficacy is limited (12, 16, 27). Therapy using diets such as the ketogenic diet reduces the ROS level and exerts protective effects on mitochondria. It is sometimes used in patients with LGS and MRC I deficiency; however, more targeted therapies are needed (17). Various strategies have been explored to advance the treatment of MRC I deficiency, including adeno-associated virus-based gene therapy, antioxidant-based clinical trials (e.g., idebenone, omaveloxolone), inhibition of mTOR, nicotinamide adenine dinucleotide modulators, mitochondrial replacement therapy, regulating mitophagy therapy, bypassing oxidative phosphorylation defects, and shifting mitochondrial DNA heteroplasmy (29, 30). In the future, targeted treatments may greatly improve outcomes for patients with LGS and MRC I deficiency.

Our study findings are significant because they present novel insights regarding MRC I deficiency characteristics and highlight MRC I deficiency as a possible etiology for LGS. One strength of our study is that the patients with LGS were enrolled based on uniform diagnostic criteria and they were subjected to treatment methods that have been used for a long period at our tertiary epilepsy and mitochondrial disease center. Furthermore, our findings include long-term follow-up data. This study provides a reliable reference for neurologists because, to the best of our knowledge, this study is the first to compare differential therapeutic outcomes between patients with LGS with and without MRC I deficiency. In addition, although LGS and MRC I deficiency are rare diseases, we were able to enroll many patients from our referral center, which enhanced the reliability of our data. However, this study is limited by its retrospective design. Although epilepsy treatment between the two groups in this study generally followed the standard LGS protocol, there were differences in treatment depending on the situation. Therefore, it is acknowledged that there are potential biases in terms of treatment methods in this study. These limitations will be strengthened in future research. In addition, genetic testing could not be performed for many patients with LGS and MRC I deficiency. As genetic testing continues to develop, the genetic etiologies of LGS accompanied with MRC I deficiency will be gradually identified in future studies. For the etiology-based treatment of LGS, developing a fundamental therapeutic agent that can alleviate MRC I deficiency is important.

## Data availability statement

The raw data supporting the conclusions of this article will be made available by the authors, without undue reservation.

## Ethics statement

This study was conducted in accordance with the tenets of the Declaration of Helsinki and the recommendations of the Institutional Review Board of Gangnam Severance Hospital, Yonsei University College of Medicine (approval number: 3-2022-0121). Written informed consent was obtained from the patients or guardians, whichever applicable. The studies were conducted in accordance with the local legislation and institutional requirements. Written informed consent for participation in this study was provided by the participants' legal guardians/next of kin.

## Author contributions

J-HN: Conceptualization, Data curation, Formal analysis, Funding acquisition, Investigation, Methodology, Resources, Validation, Visualization, Writing—original draft, Writing—review & editing. Y-ML: Conceptualization, Investigation, Supervision, Validation, Writing—review & editing.
